# Risk Factors for Renal Scarring and Deterioration of Renal Function in Primary Vesico-Ureteral Reflux Children: A Long-Term Follow-Up Retrospective Cohort Study

**DOI:** 10.1371/journal.pone.0057954

**Published:** 2013-02-28

**Authors:** Mei-Ju Chen, Hong-Lin Cheng, Yuan-Yow Chiou

**Affiliations:** 1 Department of Long Term Care, Chung Hwa University of Medical Technology, Rende Shiang, Tainan County, Taiwan, Republic of China; 2 Department of Urology, National Cheng Kung University Hospital, Tainan, Taiwan; 3 Department of Pediatrics, National Cheng Kung University Hospital, Tainan, Taiwan; 4 Institute of Clinical Medicine, National Cheng Kung University Hospital, Tainan, Taiwan; Centre Hospitalier Universitaire Vaudois (CHUV), Université de Lausanne, Switzerland

## Abstract

**Background and Purpose:**

The aim was to identify the risk factors for renal scarring and deteriorating renal function in children with primary vesico-ureteral reflux (VUR).

**Materials and Methods:**

Patients with primary VUR admitted to the National Cheng Kung University Hospital were retrospectively analyzed. The outcomes were renal scarring, assessed by technetium-99 m dimercaptosuccinic acid scanning, and renal function, assessed by estimated glomerular filtration rate. Univariate and multivariate models were applied to identify the corresponding independent predictors.

**Results:**

A total of 173 patients with primary VUR were recruited. The median age of VUR diagnosis was 10.0 months (IQR: 4.0–43.0 months). After adjusting for confounding factors, it was found that older age of VUR diagnosis (≥5 years vs. <1 year, adjusted OR = 2.78, 95% CI = 1.00–7.70, p = 0.049), higher grade of VUR (high grade [IV–V] vs. none, adjusted OR = 15.17, 95% CI = 5.33–43.19, p<0.0001; low grade [I–III] vs. none, adjusted OR = 5.72, 95% CI = 2.43–13.45, p<0.0001), and higher number of UTI (≥2 vs. 0, adjusted OR = 3.21, 95% CI = 1.06–9.76, p = 0.039) were risk factors for renal scarring, whereas a younger age of VUR diagnosis (≥5 years vs. <1 year, adjusted HR = 0.16, 95% CI: 0.05–0.51, p = 0.002), renal scarring (yes vs. no, adjusted HR = 3.66, 95% CI: 1.32–10.16, p = 0.013), and APN (yes vs. no, adjusted HR = 3.10, 95% CI: 1.05–9.14, p = 0.041) were risk factors for developing chronic kidney disease stage 2 or higher.

**Conclusions:**

Our findings expand on the current knowledge of risk factors for renal scarring and deteriorating renal function, and this information can be used to modify the management and treatment of VUR.

## Introduction

Vesico-ureteral reflux (VUR), a congenital anomaly characterized by either a unilateral or bilateral reflux of urine from the bladder to the kidney(s), has been long recognized as a major pediatric health problem[1]. VUR is frequently found in children with urinary tract infection (UTI) and acute pyelonephritis (APN), and is well known that patients with VUR are more prone to renal scarring[1], [2]. Additionally, it has been previously estimated that 15–52% of children with APN^2^ and 23–59% of children with UTI[3]–[5] exhibit renal scarring, which in turn, may lead to a worsening of renal function[2].

Previous studies have identified several risk factors for renal scarring, such as male gender, severity of VUR, history of UTI, and older age at diagnosis[5]–[9]. However, the wide ranges in the reported estimates for renal scarring suggest that there may be differences in the patient populations studied. Indeed, the incidence of renal scarring appears to be significantly higher in Asian individuals compared to other regions of the world[2]. Thus, a better understanding of the risk factors for renal scarring in Asian children would aid in the management and treatment of VUR. Furthermore, there have been limited and conflicting reports on the risk factors for deteriorating renal function, in particular the development and progression of chronic kidney disease (CKD), in children with VUR.

Thus, we evaluated the records of 173 children with primary VUR to identify risk factors for renal scarring and deteriorating renal function during long-term follow-up.

## Materials and Methods

This retrospective cohort study was based on the epidemiology and outcome studies of pediatric chronic kidney disease in Taiwan. The records of 173 patients diagnosed with primary VUR that were admitted to the National Cheng Kung University Hospital between August 1994 and July 2009 were reviewed. Patients diagnosed with primary VUR via voiding cystourethrogram were included in the analysis. Patients with VUR associated with posterior urethral valves, ectopic ureterocele, neurogenic bladder, and other obstructive uropathies below bladder were excluded from the study. The study was approved by the Institutional Review Board at the National Cheng Kung University Hospital. An informed consent was not required because this study was considered the expedited review and met the criteria for waiving informed consent based on the laws and the regulation of the Department of Health in Taiwan. Institutional Review Board of the National Cheng Kung University Hospital also approved the consent procedure and all data was analyzed anonymously.

The following clinical variables obtained from patient records were considered as potential risk factors of renal scarring and deterioration of renal function: gender, age of diagnosis of primary VUR (months), grade of primary VUR, unilateral or bilateral primary VUR, presence of a congenital urinary tract abnormality (yes/no), history of UTI, history of APN, history of renal abscess, number of UTI before and after VUR was cured, receiving prophylactic treatment (yes/no), receiving surgical treatment (yes/no), and follow-up duration until VUR was cured. VUR was graded according to International Reflux Study Committee guidelines[10]. Patients were also grouped according to the following four types of primary VUR: 1) unilateral, low grade (I–III); 2) unilateral, high grade (IV–V); 3) bilateral, low grade (I–III); and 4) bilateral, high grade (IV–V in either side).

The outcomes of interest were renal function and renal scarring. Renal function was determined from the estimated glomerular filtration rate (eGFR), which was calculated by using the Bedside Schwartz equation[11]. Renal function was classified according to the National Kidney Foundation's Kidney Disease Outcomes Quality Initiative (KDOQI) guidelines in children and adolescents[12]. Renal scarring was detected with repeated technetium-99 m labelled dimercaptosuccinic acid (DMSA) renal scintigraphy, which was performed at least 6 months after urinary tract infection[13], [14].

Other outcomes of interest were blood pressure (systolic and diastolic) and the incidence of hypertension at baseline and final follow-up, as stratified by VUR grade.

### Statistical analysis

Continuous variables are presented as median values (25–75% interquartile range, IQR), as data were not normally distributed. Categorical variables are expressed as frequencies and percentages. The demographic and clinical characteristics between patients that underwent a surgical correction for VUR and those that did not were compared by using the Wilcoxon rank-sum test for continuous variables, and the Chi-square or Fisher’s exact tests for categorical variables, where appropriate.

To determine the risk factors associated with renal scarring, the generalized estimating equation (GEE) model with a logit link function were applied to the two kidneys (right and left) within the same patient. The risk factors for renal scarring were determined via crude and adjusted odds ratios (OR) with 95% confidence intervals (CI) by using univariate and multivariate GEE models, respectively. The multivariate GEE model was constructed by using a backward selection procedure, where variables that did not improve the model fit at p<0.05 were discarded. However, gender, age of diagnosis of VUR, grade of VUR, presence of bilateral VUR and surgical correction of VUR were always forced into the model for adjustment.

Renal survival time was measured from the birth date of the patient to the date of their last follow-up. An event in the renal survival analysis was defined as the development of CKD stage 2 or higher. Patients were censored if their CKD stage was higher or equal to stage 2 (i.e. eGFR <90 ml/min/1.73 m^2^) at the last follow-up[11]. Renal survival curves were constructed by using the Kaplan-Meier method and compared by using the log-rank test to detect differences in renal survival among the 4 groups of primary VUR. The risk factors for renal survival were determined via crude and adjusted hazard ratios (HR) with 95% CI by using the Cox proportional hazard regression model. The multivariate Cox proportional hazard regression model was constructed by using the backward selection procedure, where variables that did not improve the model fit at p<0.05 were discarded. However, gender, age of diagnosis of VUR, grade of primary VUR, presence of bilateral VUR, and corrective surgery for VUR were always forced into the model for adjustment.

To evaluate the effect of primary VUR grade on blood pressure at baseline and last follow-up, analysis of covariance was performed to adjust for age at diagnosis of VUR and gender. Least-squares means and standard errors of systolic and diastolic blood pressures were calculated using general linear model adjusted for age at diagnosis of VUR and gender. Differences in proportion of patients with hypertension among the four VUR groups were compared by Fisher’s exact test.

All of the statistical analyses were performed with SAS software version 9.2 (SAS Institute Inc., Cary, NC). A two-tailed p<0.05 was considered statistically significant.

## Results

The baseline characteristics of patients are summarized in Table 1. A total of 173 patients diagnosed with primary VUR were recruited, of which 91 were male and 82 were female. The median age of VUR diagnosis was 10.0 months (IQR: 4.0–43.0 months). Among patients with unilateral VUR, 64 had a low grade VUR (i.e. I–III) and 23 had a high grade VUR (i.e. IV–V). Among those with bilateral VUR, 37 had a low grade VUR and 49 had a high grade VUR. With respect to the clinical characteristics, most patients were diagnosed with UTI (95.3%) and APN (85.9%). However, only 2.9% of patients presented with a renal abscess, and 17.3% of patients presented with a congenital urinary tract abnormality (n = 30), which included renal agenesis (n = 18), ureteropelvic junction obstruction (n = 4), VATER associated renal atrophy (n = 2), multicystic dysplastic kidney (n = 2), horseshoe kidney (n = 1), duplicated kidneys (n = 1), duplicated ureters (n = 1), renal atrophy with congenital cystic adenomatoid of lung (n = 1). Most patients (85.3%) were administrated prophylactic antibiotics initially, and approximately 41.6% of patients had undergone corrective surgery for VUR.

**Table 1:** Baseline characteristics of patients (n = 173 patients).

		Surgery for VUR^1^		
Characteristics	Total(n = 173)	No(n = 99)	Yes(n = 72)	*P*-value
Gender, n (%)				
Female	82 (47.4)	51 (51.5)	30 (41.7)	0.203^†^
Male	91 (52.6)	48 (48.5)	42 (58.3)	
Age of VUR diagnosis (month)				
Median (IQR)	10.0 (4.0–43.0)	16.0 (4.0–49.0)	7.5 (3.0–32.5)	0.024^‡^
Age group of VUR diagnosis, n (%)				
<1 year	92 (53.2)	44 (44.4)	47 (65.3)	0.026^†^
1–5 years	49 (28.3)	34 (34.3)	15 (20.8)	
≥5 years	32 (18.5)	21 (21.2)	10 (13.9)	
Grade of primary VUR, n (%)				
Unilateral, low grade (I–III)	64 (37.0)	56 (56.6)	8 (11.1)	<.0001^†^
Unilateral, high grade (IV–V)	23 (13.3)	8 (8.1)	14 (19.4)	
Bilateral, low grade (I–III)	37 (21.4)	26 (26.3)	11 (15.3)	
Bilateral, high grade (IV–V)	49 (28.3)	9 (9.1)	39 (54.2)	
UTI^2^, n (%)				
No	8 (4.7)	6 (6.1)	2 (2.8)	0.470^¶^
Yes	163 (95.3)	92 (93.9)	69 (97.2)	
APN^3^, n (%)				
No	24 (14.1)	14 (14.4)	10 (14.1)	0.949^†^
Yes	146 (85.9)	83 (85.6)	61 (85.9)	
Renal abscess, n (%)				
No	168 (97.1)	95 (96.0)	71 (98.6)	0.399^¶^
Yes	5 (2.9)	4 (4.0)	1 (1.4)	
Prophylactic antibiotics^4^, n (%)				
No	25 (14.7)	20 (20.2)	5 (7.1)	0.019^†^
Yes	145 (85.3)	79 (79.8)	65 (92.9)	
Urinary tract abnormality, n (%)				
No	143 (82.7)	86 (86.9)	56 (77.8)	0.118^†^
Yes	30 (17.3)	13 (13.1)	16 (22.2)	
Follow-up time until VUR cured^5^, n (%)				
<1 year	27 (33.3)	0 (0.0)	27 (54.0)	<.0001^†^
1–2 years	25 (30.9)	15 (48.4)	10 (20.0)	
≥2 years	29 (35.8)	16 (51.6)	13 (26.0)	
Number of UTI before VUR was cured^6^, n (%)				
0	11 (6.6)	6 (6.3)	5 (7.1)	0.811^†^
1	102 (61.1)	56 (59.0)	44 (62.9)	
≥2	54 (32.3)	33 (34.7)	21 (30.0)	
Number of UTI after VUR was cured^7^, n (%)				
0	82 (81.2)	46 (82.1)	34 (79.1)	0.803^¶^
1	12 (11.9)	7 (12.5)	5 (11.6)	
≥2	7 (6.9)	3 (5.4)	4 (9.3)	

***Notes:*** ^†^Chi-square test; ^‡^Wilcoxon rank-sum test; ^¶^Fisher’s exact test. ^1^ Two patients with missing data for surgery; ^2^ Two patients with missing data for UTI; ^3^ Three patients with missing data for APN; ^4^ Three patients with missing data for prophylactic antibiotics; ^5^ Ninety-two patients with missing data for follow-up time until VUR cured; ^6^ Six patients with missing data for number of UTI before VUR was cured; ^7^ Seventy-two patients with missing data for number of UTI after VUR was cured.

***Abbreviations:*** VUR, vesico-ureteral reflux; UTI, urinary tract infection; APN, acute pyelonephritis.

A comparison of the characteristics between patients that did and did not undergo corrective surgery for VUR revealed that those that underwent surgery were diagnosed with VUR at a significantly younger age (median (IQR): 7.5 (3.0–32.5) months vs. 16.0 (4.0–49.0) months, p = 0.024), had a more serious grade of VUR (bilateral, high grade: 54.2 vs. 9.1%, p<0.0001), were more likely to be treated with prophylactic antibiotics (92.9 vs. 79.8%, p = 0.019), and had a shorter follow-up time until VUR was cured (<1 year: 54.0 vs. 0%, p<0.0001) compared to those that did not undergo surgery. Furthermore, patients that underwent surgery were more likely to have a congenital urinary tract abnormality than those that did not undergo a surgery; however, this was not significantly different. Other characteristics were comparable between patients did and did not undergo surgery. Moreover, the association between the grade of the primary VUR and age of VUR diagnosis was investigated. Regardless of whether the VUR was unilateral or bilateral, patients with VUR diagnosed at a younger age were more likely to have a high grade VUR (IV–V) than those who were diagnosed with VUR at an older age (p = 0.009, data not shown).

Of the 173 patients, 126 had a record of undergoing a DMSA at the last follow-up. Considering both the right and left kidney, a total of 248 kidneys (4 patients with single kidney) were analyzed to determine the risk factors for renal scarring (Table 2). All grades of primary VUR, the presence of bilateral VUR, and having undergone a corrective surgery for VUR were identified as risk factors for renal scarring in univariate analysis. With respect to the grade of primary VUR, the crude ORs for renal scarring for high (IV–V) and low (I–III) grade were 12.32 (95% CI: 5.14–29.57, p<0.0001) and 4.72 (95% CI: 2.26–9.86, p<0.0001), respectively, compared to kidneys without VUR. Moreover, the crude OR for renal scarring of those with bilateral VUR was 1.98 (95% CI: 1.16–3.38, p = 0.012) compared to those with unilateral VUR. Lastly, those that underwent corrective surgery were more likely to have renal scarring than those that did not (crude OR = 2.40, 95% CI: 1.40–4.13, p = 0.002).

**Table 2:** Risk factors for developing renal scarring according to the univariate and multivariate generalized estimating equation (GEE) models (n = 248 kidneys).

	Univariate		Multivariate†	
	Crude OR (95% CI)	P-value	Adjusted OR (95% CI)	P-value
Gender				
Female	1 (reference)	--	1 (reference)	--
Male	0.88 (0.52–1.51)	0.649	0.76 (0.36–1.60)	0.472
Age group of VUR diagnosis				
<1 year	1 (reference)	--	1 (reference)	--
1–5 years	1.17 (0.64–2.17)	0.606	1.63 (0.71–3.72)	0.248
≥5 years	1.95 (0.94–4.06)	0.073	2.78 (1.00–7.70)	0.049
Grade of primary VUR				
None	1 (reference)	--	1 (reference)	--
Low grade (I–III)	4.72 (2.26–9.86)	<0.0001	5.72 (2.43–13.45)	<0.0001
High grade (IV–V)	12.32 (5.14–29.57)	<0.0001	15.17 (5.33–43.19)	<0.0001
Presence of bilateral VUR				
No	1 (reference)	--	1 (reference)	--
Yes	1.98 (1.16–3.38)	0.012	0.60 (0.28–1.31)	0.199
UTI^1^				
No	1 (reference)	--		
Yes	1.53 (0.38–6.15)	0.553		
APN^2^				
No	1 (reference)	--		
Yes	1.47 (0.70–3.11)	0.309		
Renal abscess				
No	1 (reference)	--		
Yes	0.58 (0.10–3.36)	0.543		
Prophylactic antibiotics^3^				
No	1 (reference)	--		
Yes	0.83 (0.40–1.75)	0.632		
Urinary tract abnormality				
No	1 (reference)	--		
Yes	1.33 (0.73–2.43)	0.348		
Surgery for VUR^4^				
No	1 (reference)	--	1 (reference)	--
Yes	2.40 (1.40–4.13)	0.002	2.14 (0.98–4.69)	0.057
VUR cured^5^				
No	1 (reference)	--		
Yes	0.51 (0.24–1.07)	0.076		
Follow-up time until VUR was cured^6^				
<1 year	1 (reference)	--		
1–2 years	0.84 (0.33–2.16)	0.723		
≥2 years	0.96 (0.42–2.22)	0.932		
Number of UTI before VUR was cured^7^				
0	1 (reference)	--	1 (reference)	--
1	1.73 (0.67–4.47)	0.255	1.85 (0.63–5.48)	0.264
≥2	2.62 (0.98–7.03)	0.055	3.21 (1.06–9.76)	0.039
Number of UTI after VUR was cured^8^				
0	1 (reference)	--		
1	0.61 (0.24–1.52)	0.286		
≥2	1.31 (0.32–5.39)	0.711		

***Notes:*** ^1^ n = 246; ^2^ n = 245; ^3^ n = 246; ^4^ n = 247; ^5^ n = 198; ^6^ n = 144; ^7^ n = 236; ^8^ n = 143. † In the final multivariate model, data of 235 kidneys were used.

***Abbreviations:*** VUR, vesico-ureteral reflux; UTI, urinary tract infection; APN, acute pyelonephritis.

Gender, age of VUR diagnosis, grade of primary VUR, presence of bilateral VUR, having undergone corrective surgery for VUR, and number of UTI before VUR was cured were included in the multivariate GEE model. After controlling for the other variables in the model, patients with an older age of VUR diagnosis (≥5 years vs. <1 year, adjusted OR = 2.78, 95% CI = 1.00–7.70, p = 0.049), higher grade of primary VUR (high grade vs. none, adjusted OR = 15.17, 95% CI = 5.33–43.19, p<0.0001; low grade vs. none, adjusted OR = 5.72, 95% CI = 2.43–13.45, p<0.0001), and higher number of UTI before VUR was cured (≥2 vs. 0, adjusted OR = 3.21, 95% CI = 1.06–9.76, p = 0.039) were found to be significantly associated with renal scarring.

**Figure 1:**
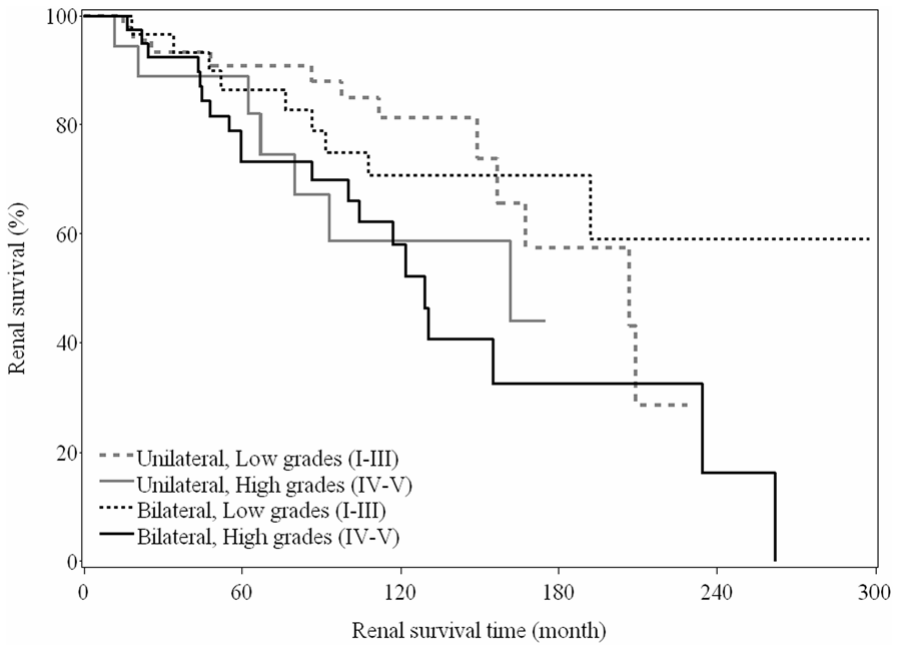
Kaplan-Meier survival curves for the development of renal failure (i.e. chronic kidney disease [CKD] stage 2 or higher) according to the grade and location (i.e. unilateral vs. bilateral) of primary vesico-ureteral reflux (VUR) (log-rank test, P  = 0.055).

Furthermore, of the 173 patients, 138 had a record of CKD stage at last visit. Patients were evaluated according to the following four groups of primary VUR: 1) unilateral, low grade (n = 48); 2) unilateral, high grade (n = 18); 3) bilateral, low grade (n = 30); and 4) bilateral, high grade (n = 42). It should be noted that patients with the presence of bilateral VUR and a high grade of VUR in either kidney were grouped in the ‘bilateral, high grade’ group. The renal survival curves for these four primary VUR groups are presented in Figure 1. However, the renal survival curves did not differ significantly among the groups (log-rank test, p = 0.055).

The age of VUR diagnosis, grade of primary VUR, and renal scarring were identified as significant risk factors for the development CKD stage 2 or higher via univariate analysis (Table 3). Compared to patients that were diagnosed with VUR at <1 year of age, those that were diagnosed with VUR at an older age had a lower risk of developing CKD stage 2 or higher (1–5 years, crude HR = 0.46, 95% CI: 0.24–0.91, p = 0.027; ≥5 years, crude HR = 0.24, 95% CI: 0.10–0.58, p = 0.002). Moreover, patients in the bilateral, high grade group had a significantly elevated risk of developing CKD stage 2 or higher (crude HR = 2.20, 95% CI: 1.06–4.54, p = 0.033) versus those in the unilateral, low grade group. Lastly, patients with renal scarring had a significantly higher risk of developing CKD stage 2 or higher (crude HR = 2.60, 95% CI: 1.02–6.65, p = 0.047) compared to those with no renal scarring.

**Table 3:** Risk factors for developing chronic kidney disease (CKD) stage 2 or higher according to univariate and multivariate Cox proportional hazard regression models (n = 138).

	Univariate		Multivariate†	
	Crude HR (95% CI)	P-value	Adjusted HR (95% CI)	P-value
Gender				
Female	1 (reference)	--	1 (reference)	--
Male	1.72 (0.97–3.05)	0.066	1.06 (0.51–2.24)	0.871
Age group of VUR diagnosis				
<1 year	1 (reference)	--	1 (reference)	--
1–5 years	0.46 (0.24–0.91)	0.027	0.43 (0.18–1.03)	0.057
≥5 years	0.24 (0.10–0.58)	0.002	0.16 (0.05–0.51)	0.002
Grade of primary VUR				
Unilateral, low grade (I–III)	1 (reference)	--	1 (reference)	--
Unilateral, high grade (IV–V)	1.85 (0.72–4.73)	0.199	0.75 (0.22–2.57)	0.644
Bilateral, low grade (I–III)	0.91 (0.38–2.18)	0.837	0.67 (0.24–1.87)	0.444
Bilateral, high grade (IV–V)	2.20 (1.06–4.54)	0.033	1.73 (0.62–4.78)	0.293
Renal scar^1^				
No	1 (reference)	--	1 (reference)	--
Yes	2.60 (1.02–6.65)	0.047	3.66 (1.32–10.16)	0.013
UTI^2^				
No	1 (reference)	--		
Yes	1.69 (0.23–12.30)	0.605		
APN^3^				
No	1 (reference)	--	1 (reference)	--
Yes	2.37 (0.93–6.07)	0.072	3.10 (1.05–9.14)	0.041
Renal abscess				
No	1 (reference)	--		
Yes	0.52 (0.07–3.76)	0.514		
Prophylactic antibiotics^4^				
No	1 (reference)	--		
Yes	1.04 (0.52–2.07)	0.916		
Urinary tract abnormality				
No	1 (reference)	--		
Yes	1.37 (0.72–2.60)	0.336		
Surgery for VUR^5^				
No	1 (reference)	--	1 (reference)	--
Yes	1.39 (0.78–2.47)	0.264	0.77 (0.35–1.70)	0.517
Follow-up time until VUR was cured^6^				
<1 year	1 (reference)	--		
1–2 years	0.73 (0.26–2.05)	0.545		
≥2 years	1.00 (0.41–2.43)	0.999		
Number of UTI before VUR was cured^7^				
0	1 (reference)	--		
1	2.24 (0.51–9.76)	0.284		
≥2	2.37 (0.53–10.62)	0.260		
Number of UTI after VUR was cured^8^				
0	1 (reference)	--		
1	0.26 (0.03–1.91)	0.184		
≥2	0.59 (0.13–2.61)	0.484		

***Notes:*** ^1^ n = 125; ^2^ n = 137; ^3^ n = 136; ^4^ n = 137; ^5^ n = 137; ^6^ n = 77; ^7^ n = 132; ^8^ n = 78. † Data of 122 patients were used in the final multivariate model.

***Abbreviations:*** VUR, vesico-ureteral reflux; UTI, urinary tract infection; APN, acute pyelonephritis.

Gender, age of VUR diagnosis, grade of primary VUR, presence of renal scarring, having undergone corrective surgery for VUR, and a history of APN were included in the multivariate Cox’s proportional hazard regression model. After controlling for the other variables in the model, a younger age of VUR diagnosis (≥5 years vs. <1 year, adjusted HR = 0.16, 95% CI: 0.05–0.51, p = 0.002), renal scarring (yes vs. no, adjusted HR = 3.66, 95% CI: 1.32–10.16, p = 0.013), and APN (yes vs. no, adjusted HR = 3.10, 95% CI: 1.05–9.14, p = 0.041) were significant risk factors for the development of CKD stage 2 or higher.

Complete data on blood pressure measurements are available for 152 subjects at baseline and 101 subjects at last follow-up. The proportion of patients with hypertension at baseline ranged from 0.0 to 5.0%, whereas the proportion of patients with hypertension at last follow-up ranged from 7.1 to 33.3%. Regardless of the timing of assessment (baseline or last follow-up), there were no significant differences in the incidence of hypertension among the four VUR grade groups (Table 4). Further, there were no significant differences in either systolic or diastolic blood pressure among the four VUR grade groups after adjusting for age at VUR diagnosis and gender (Table 4).

**Table 4:** Blood pressure and hypertension incidence by primary VUR grade at baseline (n = 152) and last follow-up (n = 101).

	Primary VUR grade				
	Unilateral,Low grade(I–III)	Unilateral,High grade(IV–V)	Bilateral,Low grade(I–III)	Bilateral,High grade(IV–V)	P-value
***Baseline***					
Systolic blood pressure (mmHg)	n = 58	n = 20	n = 30	n = 44	
LS-mean (SE)^1^	96.36 (1.31)	92.32 (2.33)	98.50 (1.84)	98.13 (1.53)	0.149^†^
Diastolic blood pressure (mmHg)					
LS-mean (SE)^1^	55.47 (1.37)	55.73 (2.42)	58.62 (1.92)	57.06 (1.60)	0.579^†^
Hypertension, n (%)					
No	58 (100.0)	19 (95.0)	30 (100.0)	42 (95.5)	0.124^¶^
Yes	0 (0.0)	1 (5.0)	0 (0.0)	2 (4.6)	
***Last follow-up***					
Systolic blood pressure (mmHg)	n = 36	n = 14	n = 21	n = 30	
LS-mean (SE)^1^	95.17 (1.85)	90.94 (3.03)	98.12 (2.34)	97.78 (1.97)	0.199^†^
Diastolic blood pressure (mmHg)					
LS-mean (SE)^1^	56.07 (1.82)	53.06 (2.98)	58.86 (2.31)	57.13 (1.94)	0.474^†^
Hypertension, n (%)					
No	32 (88.9)	13 (92.9)	16 (76.2)	20 (66.7)	0.085^¶^
Yes	4 (11.1)	1 (7.1)	5 (23.8)	10 (33.3)	

***Notes:*** ^†^ Analysis of covariance; ^¶^ Fisher’s exact test; ^1^ LS-means and SE were calculated using a general linear model adjusted for age at VUR diagnosis and gender.

***Abbreviations:*** VUR, vesico-ureteral reflux; LS, least squares; SE, standard error.

## Discussion

In the present study, we investigated the potential risk factors for renal scarring and deteriorating renal function in pediatric patients with unilateral or bilateral primary VUR of all grades. After adjusting for confounding factors, it was found that an older age of VUR diagnosis, higher grade of VUR, and higher number of UTI were risk factors for renal scarring, whereas a younger age of VUR diagnosis, renal scarring, and a history of APN were risk factors for developing CKD stage 2 or higher.

It is well known that patients with VUR are more prone to renal scarring. However, the risk factors for renal scarring in Asian children with VUR, where the incidence of renal scarring is the highest in the world[2], have not been identified. Soylu et al. found that male gender, age ≥27 months in girls, grades IV–V of VUR, and the presence of previous renal scarring were independent predictors of renal scarring[9]. In another study of children with primary familial VUR, grade of VUR, history of UTI, and older age at diagnosis were found to be independent risk factors for renal scarring^6^. Furthermore, in a Southeast Asian population, Vachvanichsanong et al. demonstrated that a high-grade unilateral VUR, an age of diagnosis >5 years, and male gender were the most significant risk factors for renal scarring[5]. Similarly, Silva et al. identified grades III–V of VUR, age at diagnosis, unilateral reflux, and male gender as risk factors for renal scarring[8]. Lastly, in a retrospective study on children with a first time UTI, the relative risk of renal damage was found to be significantly increased in patients with grade II or higher VUR[7]. Corroborating most previous findings, in the present study, we identified that an older age of VUR diagnosis (≥5 years), higher grade of VUR, and higher number of UTI were risk factors for renal scarring.

It is not surprising that the severity of renal scarring is associated with the severity of VUR. Yoneda et al. demonstrated that, if the VUR grade increased by one grade, the risk for renal damage was 3.5-times higher[6]. Furthermore, it was previously reported that patients with high-grade VUR are 4–6 times more likely to have renal scarring than those with low-grade reflux, and 8–10 times more likely than those without VUR[5]. In the present study, patients with low (I–III) and high (IV–V) grade VUR were found to have a 5.72 and 15.17 fold increased risk of renal scarring, respectively. In addition to VUR severity, the age at diagnosis is also an important risk factor. It was previously determined that an increase of 1 year in the age of diagnosis corresponds with a 1.2-fold greater risk of renal scarring[6]. We found that there is a 2.78 fold higher risk of renal scarring if diagnosis was made at ≥5 years. Thus, based on these findings, efforts to prevent and/or reduce renal scarring should be directed towards a rapid diagnosis and treatment of VUR.

Few reports have investigated the risk factors for deteriorating renal function associated with VUR. According to a comprehensive epidemiological study conducted in Italy (i.e. ItalKid Project), VUR *per se* was found to be the single leading cause of CKD in children, accounting for roughly 26% of the cases[15]. However, this is in contrast with the findings of the North American Pediatric Renal Trials and Collaborative Studies (NAPRTCS) Registry, in which only 8.5% of patients had VUR as the cause of CKD[16]. In addition to the prevalence of CKD among VUR patients, findings regarding the predictive risk factors for the development and progression of CKD in children with VUR have been conflicting. Caione et al. concluded that bilateral high grade VUR and serum creatinine levels >6.0 mg/L in the first year of life are significant risks for the development of CKD[17]. Conversely, Silva et al. determined that children aged >24 months at diagnosis, grade V VUR, bilateral renal damage, and a delay in the diagnosis of VUR of >12 months after UTI were independent predictors of CKD[1], [18]. Moreover, a report by Novak et al. on data from the NAPRTCS suggested that older age, higher CKD stage, and history of UTI are significant risk factors for CKD progression in children with VUR[16]. Interestingly, in the present study, the risk factors for deteriorating renal function were completely different from those identified in previous studies. Specifically, we found that patients with a younger age of VUR diagnosis, renal scarring, and history of APN had a significantly higher risk of developing CKD stage 2 or higher. These discrepancies may be explained, in part, by differences in patient populations (i.e. ethnic differences), difference of medical insurance and groupings used in data analyses. Thus, further studies in Asian populations are warranted to confirm the risk factors for CKD associated with VUR.

In addition to expanding on the knowledge regarding the risk factors, we found that a greater proportion of patients with a younger age of VUR diagnosis, more severe VUR (i.e. bilateral, high grade VUR), and previously treated with prophylactic antibiotics underwent corrective surgery for VUR than those that did not. These findings may be explained by the fact that traditional management for VUR consists of long-term use of prophylactic antibiotics with surgery reserved for patients with recurrent UTI or worsening renal scarring[16]. However, the usefulness of this treatment paradigm has been recently questioned[19]. First, physicians should be cautious in using prophylactic antimicrobials for UTI, as treatment is not associated with a decreased risk of recurrent UTI, but associated with an increased risk of resistant infections that further exacerbate the condition[20]. Furthermore, a 10-year follow-up study on patients with grades III–IV VUR randomized to either surgical or medical treatment reported that there was no difference in renal scarring[7]. Lastly, a meta-analysis of randomized controlled trials found that it was unclear whether antibiotics or surgery for VUR were more beneficial[7].

We found that a relatively high proportion of patients had hypertension at final follow-up. Although there were no significant differences between VUR grades, the proportion of patients with hypertension was particularly high for those who had bilateral low (23.8%) and high (33.3%) grade VUR. These rates of hypertension are similar, but slightly higher, than those reported in a previous study, in which 15% of pediatric patients with primary VUR had hypertension at 21 years of age [21]. The findings from our study and the previous study [21] suggest that the blood pressure of patients with VUR should be regularly monitored, and appropriate medication prescribed as necessary to prevent / control hypertension.

The main strengths of this study are that this study was the first to assess the risk factors for renal scarring and deteriorating renal function in an Asian pediatric population, and renal scarring was evaluated with the more sensitive DMSA scan[13]. While the present study builds on the current foundation knowledge of VUR, there are a few limitations that need to be mentioned, including that this was a single-site, retrospective study with a small sample size and different follow-up durations.

## Conclusions

In children with primary VUR, older age of VUR diagnosis, higher grade of VUR, and higher number of UTI were risk factors for renal scarring, whereas a younger age of VUR diagnosis, renal scarring, and a history of APN were risk factors for deteriorating renal function. A better understanding of the risk factors for renal scarring and deteriorating renal function can be useful in tailoring the management and therapeutic approach for VUR. Additionally, the identification of risk factors may provide etiological and pathogenic insights on the disease, as well as provide novel targets for the development of new therapies. Finally, we suggest that children diagnosed with VUR should be followed-up during adulthood, particularly if they have risk factors for CKD.
